# Selecting for Learning Potential: Is Implicit Learning the New Cognitive Ability?

**DOI:** 10.3390/jintelligence10020024

**Published:** 2022-04-15

**Authors:** Luke M. Montuori, Lara Montefiori

**Affiliations:** 1 Arctic Shores Ltd., Manchester M2 3AW, UK; 2 Department of Psychology & Language Sciences, University College London, London WC1H 0AP, UK; lara.montefiori.16@ucl.ac.uk

**Keywords:** implicit learning, workplace selection, cognitive ability, psychometric testing, behavioural task design

## Abstract

For decades, the field of workplace selection has been dominated by evidence that cognitive ability is the most important factor in predicting performance. Meta-analyses detailing the contributions of a wide-range of factors to workplace performance show that cognitive ability’s contribution is partly mediated by the learning of task-relevant skills and job-specific declarative knowledge. Further, there is evidence to suggest that this relationship is a function of task complexity, and partially mediated by learning performance in workplace induction and training activities. Simultaneously, evidence is mounting that stable individual differences in implicit learning exist, which are at least partially independent of traditional measures of intelligence. In this article we provide an overview of recent advances in our understanding of implicit learning, outline some of the advantages offered by its measurement, and highlight some of the challenges associated with its adoption as a measure of interest.

## 1. Psychological Constructs and the Workplace

The history of advances in psychometrics runs parallel to the applied use of the psychological constructs of interest, particularly within organisational contexts. Notably, some of the earliest uses of psychometric measures in modern society were concerned with the selection of military personnel (Knight 2017). Since then, the study of individual differences in organisational settings across a range of sectors has revealed that psychological predictors of workplace performance are many and varied. Individual differences in personality provide information about likely behaviours when confronted with various work-specific scenarios and interpersonal relationships, and overall inform workplace outcomes (Mount et al. 1998). Individuals’ motivations provide information about the kinds of tasks or roles an individual will find themselves attracted to, and amount of discretionary effort likely to be expended in pursuing job-specific goals (Nye et al. 2012). Measures of integrity provide insight into the extent to which an individual is likely to engage in behaviours that are counterproductive to organisational goals (Ones et al. 1995). Additionally, individual differences in the ability to acquire task-relevant knowledge skills are key predictors of workplace outcomes (Colquitt et al. 2000; Danner et al. 2011).

Although there is a wide variety of ways in which disparate psychological constructs have been shown to contribute towards workplace outcomes, no construct has been shown to be so impactful across such a breadth of scenarios as cognitive ability (Schmidt and Hunter 2004). The extent to which learning ability and cognitive ability can be dissociated, and to which learning can be considered both a unique ability and key mechanism through which cognitive ability impacts workplace performance, is the subject of the present article.

## 2. Cognitive Ability and Workplace Performance

In a seminal piece of research within the occupational psychology literature, Schmidt and Hunter (1998) compared the relative validity and utility of various personnel selection methods. Their meta-analysis, compiling results from across a broad range of industries and roles, and using a variety of outcome measures (supervisor ratings or objective metrics of workplace and training performance), identified and ranked the strongest predictors of workplace outcomes. Although tests of individual differences in personality, knowledge, and experience bore some relation to workplace performance, cognitive ability measures were amongst the most predictive, second only to work sample tests.

The literature supporting the view that cognitive ability is a strong predictor of workplace outcomes, and thus useful in the context of personnel selection, has continued to grow (Ones et al. 2017). These findings have also been seen to generalise cross-culturally (Salgado et al. 2003). Cognitive ability can also be seen as a reliable but complex contributor to workplace performance. For example, cognitive ability contributes to workplace attitudes (Anglim et al. 2019), and interacts variably with other individual differences, such as conscientiousness, depending on task and context (Harris-Watson et al. 2022). While these findings are of interest due to the insight they provide into human behaviour generally, they are also of great consequence. Organisations are highly motivated to apply the findings of this field of research, as the use of hiring methods with greater predictive validity has tangible outcomes, such as increased productivity and increased monetary value of output (Hunter et al. 1990).

Despite the consistency with which these effects have been reported they have not gone unchallenged, and the use of cognitive ability assessment within personnel selection is not without issue. Tests of cognitive ability sometimes provide differing results on the basis of race, sex, and ethnicity (Neisser et al. 1996). Thus, relying heavily on cognitive ability scores in selection decisions can contribute to adverse impact, where members of one group are disproportionately selected over another. Motivations for reducing adverse impact are varied, but can be justified on the basis of legality, morality, and productivity (Burgoyne et al. 2021). For example, increased diversity is positively associated with greater decision-making capability and effectiveness (De Dreu and West 2001; McLeod et al. 1996). This is at least partly attributable to increased creativity and innovation, which is in turn facilitated by individuals interacting with a wider breadth of perspectives. From a moral perspective, organisations may value a workforce that is more representative of the population at large (Sackett et al. 2001), and may be particularly motivated by feelings of justice to improve diversity and representation (Moses 2010). The continued use of cognitive ability tests is, as a result of these perspectives, in a difficult position. Their role in selection processes has the potential to disadvantage some populations. Meanwhile, other populations are disproportionately selected, and thus overrepresented in management positions and organisations more generally (Ng and Sears 2010).

An additional and perhaps more fundamental criticism of the use of tests of cognitive ability comes from the characterisation of its relationship with learning. For some time, cognitive ability has been equated with ‘learning ability’. Spearman (1927) described differences in cognitive ability, or the positive manifold of intelligence, as being differences in the “eduction of relations and correlates”, and the view that cognitive ability is synonymous with ‘ability to learn’ remains pervasive within the psychological literature (Schmidt and Hunter 2003; Mackintosh 2004). However, despite this widespread view, there are those who have observed that measures of cognitive ability do not measure ‘learning ability’. For example, Richardson and Norgate (2015) highlight that measures of cognitive ability are more likely to reflect the availability or non-availability of a specific type of learned experience (something which is also true for non-verbal, or supposedly ‘culture-free’ tests of cognitive ability). The view that cognitive ability might reflect differences in educational opportunities is supported, at least partially, by evidence that experience in education contributes to increases in cognitive ability (Ceci 1991; Ritchie and Tucker-Drob 2018). The perspective that cognitive ability measures are an imperfect assessment of learning ability has also been echoed by Kuhn (2016), who points out that within tests of cognitive ability, there is no requirement for test-takers to learn anything. At best, Kuhn argues, these measures indirectly tap into learning processes. Meanwhile, individual differences in learning, far from being the result of differences in cognitive ability, are likely to be the result of a range of additional constructs such as procedural skills, dispositional factors, monitoring and managing processes (i.e., executive function), and self-regulation.

## 3. Cognitive Ability and Training Outcomes

Despite criticism of the idea that cognitive ability is a measure of learning ability, there is significant evidence that cognitive ability is at least predictive of learning outcomes. For example, higher cognitive ability has been shown to predict better educational attainment as measured by markers of graduate student performance, while also predicting subsequent transition to and performance in occupational settings (Kuncel et al. 2004). Within the workplace specifically, early research reported by Schmidt et al. (1986) identified cognitive ability as the best predictor of acquisition of job knowledge (a relationship that remained consistent even at increased levels of job experience; (Schmidt et al. 1988)). This general observation, that cognitive ability predicts training outcomes, is well-substantiated within the literature (Ree and Earles 1991; Schmidt et al. 2008; Oakes et al. 2001; Van Iddekinge et al. 2018). Causal analyses of this relationship have suggested that improved workplace outcomes result from two effects of cognitive ability: on-the-job problem solving, and the acquisition of job-relevant skills and knowledge (Schmidt 2002). Further, this relationship appears to be dependent on the information processing requirements of the tasks considered. For example, task complexity has been shown to moderate the extent to which cognitive ability predicts training outcomes, with the relationship being stronger for higher complexity tasks (Salgado and Moscoso 2019).

It has also been suggested that ability requirements vary both with task type and during the various stages of skill acquisition. In observing individual learning performance on tasks varying in consistency and complexity, Ackerman (1988) identified three phases of skill-acquisition. These three phases, declarative, knowledge, compilation, and procedural, were found to vary in terms of the extent to which they were dependent on cognitive ability. Specifically, higher intelligence was associated with faster acquisition of declarative knowledge, and greater perceptual speed was associated with improved compilation ability. Once a task was learned and performance highly automated, cognitive ability became less important than psychomotor abilities. Ackerman also identified variations in task type that differed in their reliance on cognitive ability. Inconsistent tasks, defined as those with no invariant rules or components, most benefited from greater cognitive ability. Consistent with this, Murphy (1989) describes a model in which cognitive ability is important primarily during skill development and the performance of unfamiliar tasks.

Despite the lack of clarity around whether cognitive ability should be considered synonymous with learning, it is clear at least that cognitive ability can be considered a predictor of learning outcomes and is differentially relevant during various stages of skill acquisition. However, this says little about the relationship between learning outcomes themselves and overall workplace performance. In examining the relationship between individual factors and ultimate determinants of workplace performance, Colquitt et al. (2000) identified that learning was a key predictor of workplace performance. In their meta-analysis of workplace learning, individual characteristics, and workplace performance, they demonstrated that the relationship between cognitive ability and workplace performance is mediated largely by factors associated with training; specifically, the acquisition of role-relevant skills, along with post-training feelings of self-reported efficacy. Although workplace performance was also seen to be affected by demographic factors, personality traits, and motivations, the contribution of cognitive ability to predicting workplace performance was largely through the prediction of skill acquisition.

An additional piece of evidence for the relationship between learning and workplace outcomes comes from Danner et al. (2011). In comparing the relative predictive contributions of cognitive ability, complex decision making, and implicit learning, to objective measures of professional success, both complex decision making and implicit learning were identified as being unique constructs, separate from cognitive ability. Additionally, each of these constructs were shown to predict success in the workplace. In the case of implicit learning, however, there appeared to be no additional prediction of success beyond that which was already accounted for by cognitive ability, which would indicate that implicit learning as a construct did not uniquely predict workplace outcomes. However, as Danner et al. note, psychometric issues such as test length would have hindered their ability to identify this relationship even if it were there.

## 4. Implicit Learning

Implicit learning, statistical learning, and implicit statistical learning are terms used in distinct but overlapping fields of inquiry into the unconscious acquisition of the statistical structure of perceived information. This process of acquisition is a general and universal one, and has been described as playing a foundational role in the acquisition of new abstract information (Reber 1989). Typically characterised as a set of processes that are automatic, associative, nonconscious, and unintentional, implicit learning is considered distinct from more intentional types of learning thought to be associated with executive functioning and working memory. Although there is some evidence for the existence of overlapping functionality between explicit and implicit learning processes (Ashby et al. 2003; Knowlton et al. 1994), there remains a great deal of support for their distinctiveness, both neurobiologically and on behavioural measures (Gabrieli 1998). Further, implicit and explicit learning processes have been shown to be differentially associated with a number of important outcomes, including various measures of intelligence, academic achievement, and self-reported personality (Gebauer and Mackintosh 2007; Kaufman et al. 2010).

### 4.1. Implicit Learning Predictions

The measurement of implicit learning is of great interest to researchers in a variety of domains, partly due to its association with learning outcomes and its potential distinctiveness from cognitive ability. Implicit learning, as measured by a variety of tasks, has been shown to be differentially associated with measures of cognitive ability. For instance, in manipulating the explicit/ implicit nature of the learning task, Gebauer and Mackintosh (2007) were able to vary the extent to which learning was associated with cognitive ability. The learning task, when delivered in a way that involved explicit processes, was more strongly associated with cognitive ability. When delivered in a way that involved implicit learning processes, scores on the learning task were not associated with cognitive ability. The same pattern of results, albeit with there being a weak cognitive ability/implicit association, was reported in a series of structural equation models developed by Kaufman et al. (2010). Independently, implicit learning was found to be unrelated to measures of working memory. Kaufman et al. (2010) also report associations between implicit learning and personality or outcome measures. Implicit learning was higher in individuals who self-reported as being more intuitive, open to experience, and impulsive (see also Christensen et al. 2018).

Another key finding by Kaufman et al. (2010) was that implicit learning uniquely predicted foreign language attainment. This is largely consistent with another body of findings, in which implicit learning has been associated with a variety of language-specific skills. This is especially true within the context of childhood language acquisition, with implicit learning predicting better syntactic acquisition (Kidd 2012), comprehension (Kidd and Arciuli 2016), and reading ability (von Koss Torkildsen et al. 2019). The link between implicit learning and language is not specific to children though, with implicit learning scores predicting second language acquisition in both children and adults (Granena 2012). Nor does implicit learning just predict basic language skills, but also metaphorical and abstract thinking (Drouillet et al. 2018). In view of these and similar results, it has been suggested that implicit learning is a key requirement for language learning, perception, categorization, segmentation, transfer, and generalisation (Frost et al. 2015). Finally, there is some statistical evidence that implicit learning and decision making are separate and distinct contributors to the prediction of workplace performance (Danner et al. 2011).

In addition to making a distinct contribution to the prediction of a range of relevant outcomes, implicit learning has been shown to be uniquely protected in a variety of circumstances. For instance, in one examination of the differential impact of affective states on explicit and implicit processes, Rathus et al. (1994) demonstrated that tasks dependent on explicit processes were susceptible to performance deficits resulting from test anxiety. In contrast, performance on implicit learning tasks remained unaffected by anxiety levels. Rathus et al. attribute this dissociation to the *robustness principle*, which proposes that implicit and automatic processes should be more protected from disorder and disruption than explicit and conscious ones (see Reber et al. 1991).

Implicit learning not only appears to be unaffected by affective states, but has also been shown to be protected in individuals with a number of developmental and learning disorders. For example, there is evidence that implicit learning deficits are not a feature of autism spectrum disorders (Brown et al. 2010; Foti et al. 2015). Nor is implicit learning significantly impacted in dyslexia (Inácio et al. 2018). Implicit learning thus represents a construct which is key to learning, distinct from cognitive ability, and at least partially associated with workplace outcomes. It is differentially affected in various disorders and learning disabilities, offering a unique source of information about individuals’ likely workplace performance, as separate from measures of cognitive ability.

### 4.2. Measuring Implicit Learning

Tasks purporting to measure implicit learning vary widely in terms of the stimuli used, behaviours measured, and the types of statistical structures involved. In reviewing the literature relating to implicit learning, Cleeremans et al. (1998) identify a number of commonalities between behavioural tasks seeking to measure implicit learning. According to them, situations in which implicit learning can be said to have occurred involve the following: (1) exposure to some complex rule-governed environment under incidental learning conditions; (2) a measure that tracks how well subjects can express their newly acquired knowledge about this environment through performance on the same or on a different task; and (3) a measure of the extent to which subjects are conscious of the knowledge they have acquired. Thus, while research into the nature and characteristics of implicit learning features tasks that rely on multiple modalities, the basic structure of these tasks remains the same. Research participants are incidentally exposed to statistical regularities, changes in behaviour are measured, and participants report on the extent to which they were conscious of those regularities. The following represent a selection of commonly used behavioural tasks used to measure implicit learning.

#### 4.2.1. Artificial Grammar Learning

One early example of an experimental paradigm measuring implicit learning is the artificial grammar learning (AGL) task (Reber 1967). In this task a synthetic set of grammar rules govern whether letters or symbols can be associated with each other, and under what circumstances their association is considered to be grammatically correct. After a learning or exposure phase, participants are presented with grammatical and ungrammatical strings and asked to judge the grammatical correctness of these target items. Here, the successful identification of grammatically correct strings is taken as evidence of the implicit acquisition of the statistical rules governing string production.

#### 4.2.2. Serial Response Time Task

An alternative experimental paradigm used to measure implicit learning is the serial response time task (Nissen and Bullemer 1987). In serial response tasks, participants view a screen on which stimuli are presented sequentially, in one of multiple locations. Each stimulus location is associated with a unique response, and participants are instructed to give the associated response with each stimulus appearance. Critical to the measurement of learning is the fact that embedded within the sequence of stimulus appearances are a number of smaller, repeated sequences. In this task, decreasing response times on repeatedly presented stimulus sequences are interpreted as resulting from implicit learning processes.

#### 4.2.3. Implicit Category Learning

Modelled in part on the prototype distortion category learning task (Posner and Keele 1968), the implicit category learning task (Fried and Holyoak 1984; Kalra et al. 2019) measures the formation of category judgements following repeated exposure to differing category exemplars. Task participants are asked to classify abstract visual stimuli into one of two hidden unknown categories. In this task, greater accuracy of category choices and improved awareness of category definitions are interpreted as resulting from implicit learning processes.

### 4.3. Individual Differences in Implicit Learning

The treatment of implicit learning as a distinct psychological construct represents a measurement opportunity in a range of areas, not least within the organisational application of psychometrics. Despite the unique predictions made by implicit learning measures, it is only recently that their value as predictors of stable individual differences has become apparent. To date, measures of implicit learning have yet to be used extensively within this context, despite the prolific use of cognitive ability assessments. This imbalance can be attributed to the fact that (a) stable individual differences in implicit learning are a relatively new observation, and (b) measures of implicit learning are more technically challenging to develop and administer in organisational contexts than other, more frequently used measures (i.e., multiple choice questions and Likert scales). Thus, the application of implicit learning measures within organisational settings depends on addressing these outstanding issues. In the first instance, identifying which design features of implicit learning measures most reliably produce stable individual differences. In the second instance, creating such measures in contexts that allow for their widespread deployment in organisational settings.

Much of the early research into the nature of implicit learning—its structure, qualities, and mechanisms—was focused primarily on the analysis of group level differences. Suggestions that there are stable individual differences have not always been fully supported. Studies have compared multiple measures and not observed cross-tasks correlations (Gebauer and Mackintosh 2007). Criterion validation methods in which implicit learning tasks are shown to be correlated with stable traits (such as IQ; Kaufman et al. 2010) have been taken as evidence for the stability of implicit learning generally. However, demonstrations of test–retest reliability have been inconsistent. Kalra et al. (2019) recently set out to address this issue across a variety of disparate implicit learning tasks; serial response time, artificial grammar learning, probabilistic classification, and category learning. Moderate test–retest reliability was seen in all tasks except for the artificial grammar learning task. Kalra et al. were also able to replicate a number of previous findings; specifically, the dissociation between implicit learning and various measures of explicit awareness, and the dissociation between implicit learning and conventional measures of intelligence. Regarding the lack of test–retest reliability in the case of the artificial grammar learning tasks, Kalra et al. provide a number of interpretations. First, it is suggested that explicit awareness contaminates performance at the second time point. Secondly, differences in test–retest reliability between tasks are suggested to be the result of differing mechanisms underlying the type of implicit learning measured by each task. However, there is reason to believe that these may not be sufficient to explain the lack of identifiable individual differences on the artificial grammar learning task, and that methodological issues may be more relevant.

Siegelman et al. (2017) identify a variety of critical methodological issues in the measurement and study of implicit learning. Specifically, they describe methodological and task design issues arising from historical focus on group differences research. Put another way, tasks developed thus far have been successfully designed to identify group differences in implicit learning. The same task design principles that have allowed for these observations are considered to be barriers to the identification of individual differences. Key among these principles, according to Siegelman et al., are the lack of trial numbers. For example, during the familiarisation phase of some implicit learning tasks, exposure is often limited to eight unique combinations or fewer. Absent the duplication of test stimuli, Frost et al. (2015) demonstrated in their simulation that such a test length lacks the required sensitivity to detect differences in individuals whose probability of detection is 0.6 and 0.8, respectively. In contrast, increasing trial numbers to at least 16 or 32 drastically minimises measurement error and increases task sensitivity. As a result of this, Siegelman et al. suggest that in tasks of insufficient length performance effects are potentially driven by spurious, chance responding, and highlight that good tests of individual differences must have a large number of trials, with minimal number of trial repetitions.

One additional and related issue is that implicit learning tasks feature items of equal difficulty, which Siegelman et al. address through the application of a modern psychometric approach; specifically, item response theory, in which items are constructed to have varying difficulties, and associated with differential response patterns on the basis of candidate ability. In addressing these issues, the authors demonstrate that adequate psychometric properties are attainable in these tasks, including artificial grammar learning. Finally, it is worth noting that these same impediments were reported by Danner et al. (2011). In reference to their observation that implicit learning did not uniquely predict workplace outcomes over and above cognitive ability, Danner et al. suggest that unsystematic measurement error may have obscured such a relationship, highlighting the need for greater sensitivity in implicit learning tasks.

## 5. Conclusions

Individuals’ ability to learn about the statistical regularities of stimuli in their environments has a profound impact on their acquisition of new skills and successful navigation of their environments. To the extent that successful skill acquisition predicts enhanced workplace outcomes, individual differences in implicit learning have the potential to predict individuals’ workplace potential.

Until recently, there has been little evidence for reliable individual differences in implicit learning. There have since been multiple independent demonstrations that tasks measuring implicit learning are able to achieve levels of psychometric quality required for their use in informing organisational outcomes. Deployment within organisational settings is, however, a separate challenge. Unlike traditional psychometric assessments, such as personality assessments or instruments measuring cognitive ability, measures of implicit learning are highly dependent on behavioural data. In this regard though, they are well suited to adaptation within the context of ‘*theory-driven game-based assessment*’ (Landers et al. 2021). This contemporary approach to instrument development is unique in its combining of design principles and psychometric practice, with psychological constructs previously confined to the laboratory being the ideal subjects of business-to-business software development processes. Thus, despite the additional complexity involved in the development and large-scale deployment of behavioural tasks, there is indeed both scope and an existing framework for constructs such as implicit learning to be considered within the context of organisational decision making. Although learning and developmental efforts within organisations are likely to cross a range of learning types, improving learning outcomes is a key strategic focus of organisations (Noe et al. 2014), and the reliable measurement of stable individual differences in learning ability is a key part of this endeavour (Kuhn 2016). Here, it is also worth highlighting that prominent models accounting for knowledge generation and dissemination already distinguish the role that implicit learning processes have to play in organisational learning (Nonaka and Toyama 2003). Furthermore, in an environment where there are significant reasons to move away from traditional measures of intelligence and cognitive ability as selection criteria, selecting on the basis of individual differences in implicit learning represents a potential paradigm shift in the way organisations select employees.

It is important to stress that despite the potential represented by tasks of implicit learning, there remain challenges that impede their adoption within personnel selection. Indeed, a number of these challenges continue to be the focus of research in the implicit learning literature. For instance, although there is significant evidence demonstrating the dissociation between cognitive ability and implicit learning, much is still unknown about the exact nature of the relationship between the two constructs. The structure of implicit learning processes also remains uncertain, with a number of outstanding questions around whether disparate tasks measure a single underlying learning construct, or many.

Finally, given the current absence of robust implicit learning measures from organisational contexts, the extent of the relationship between this construct and workplace outcomes remains unclear. Thus far, evidence would suggest that this is due to the lack of appropriately constructed psychometric measures of implicit learning (Danner et al. 2011; Siegelman et al. 2017). However, it is hoped that the current paper draws attention to this notable absence, and assists in advancing the more widespread measurement of individual differences in learning ability in the workplace and beyond.

## Data Availability

Not applicable.

## References

[B1-jintelligence-10-00024] Ackerman Phillip L. (1988). Determinants of individual differences during skill acquisition: Cognitive abilities and information processing. Journal of Experimental Psychology: General.

[B2-jintelligence-10-00024] Anglim Jeromy, Sojo Victor, Ashford Linda J., Newman Alexander, Marty Andrew (2019). Predicting employee attitudes to workplace diversity from personality, values, and cognitive ability. Journal of Research in Personality.

[B3-jintelligence-10-00024] Ashby F. Gregory, Noble Sharon, Filoteo J. Vincent, Waldron Elliott M., Ell Shawn W. (2003). Category learning deficits in Parkinson’s disease. Neuropsychology.

[B4-jintelligence-10-00024] Brown Jamie, Aczel Balazs, Jiménez Luis, Kaufman Scott Barry, Grant Kate Plaisted (2010). Intact implicit learning in autism spectrum conditions. The Quarterly Journal of Experimental Psychology.

[B5-jintelligence-10-00024] Burgoyne Alexander P., Mashburn Cody A., Engle Randall W. (2021). Reducing adverse impact in high-stakes testing. Intelligence.

[B6-jintelligence-10-00024] Ceci Stephen J. (1991). How much does schooling influence general intelligence and its cognitive components? A reassessment of the evidence. Developmental Psychology.

[B7-jintelligence-10-00024] Christensen Alexander P., Kenett Yoed N., Cotter Katherine N., Beaty Roger E., Silvia Paul J. (2018). Remotely close associations: Openness to experience and semantic memory structure. European Journal of Personality.

[B8-jintelligence-10-00024] Cleeremans Axel, Destrebecqz Arnaud, Boyer Maud (1998). Implicit learning: News from the front. Trends in Cognitive Sciences.

[B9-jintelligence-10-00024] Colquitt Jason A., LePine Jeffrey A., Noe Raymond A. (2000). Toward an integrative theory of training motivation: A meta-analytic path analysis of 20 years of research. Journal of Applied Psychology.

[B10-jintelligence-10-00024] Danner Daniel, Hagemann Dirk, Schankin Andrea, Hager Marieke, Funke Joachim (2011). Beyond IQ: A latent state-trait analysis of general intelligence, dynamic decision making, and implicit learning. Intelligence.

[B11-jintelligence-10-00024] De Dreu Carsten K. W., West Michael A. (2001). Minority dissent and team innovation: The importance of participation in decision making. Journal of Applied Psychology.

[B12-jintelligence-10-00024] Drouillet Luc, Stefaniak Nicolas, Declercq Christelle, Obert Alexandre (2018). Role of implicit learning abilities in metaphor understanding. Consciousness and Cognition.

[B13-jintelligence-10-00024] Foti Francesca, De Crescenzo Franco, Vivanti Giacomo, Menghini Deny, Vicari S. (2015). Implicit learning in individuals with autism spectrum disorders: A meta-analysis. Psychological Medicine.

[B14-jintelligence-10-00024] Fried Lisbeth S., Holyoak Keith J. (1984). Induction of category distributions: A framework for classification learning. Journal of Experimental Psychology. Learning, Memory, and Cognition.

[B15-jintelligence-10-00024] Frost Ram, Armstrong Blair C., Siegelman Noam, Christiansen Morten H. (2015). Domain generality versus modality specificity: The paradox of statistical learning. Trends in Cognitive Sciences.

[B16-jintelligence-10-00024] Gabrieli John D. E. (1998). Cognitive neuroscience of human memory. Annual Review of Psychology.

[B17-jintelligence-10-00024] Gebauer Guido F., Mackintosh Nicholas J. (2007). Psychometric intelligence dissociates implicit and explicit learning. Journal of Experimental Psychology: Learning Memory and Cognition.

[B18-jintelligence-10-00024] Granena Gisela (2012). Age Differences and Cognitive Aptitudes for Implicit and Explicit Learning in Ultimate Second Language Attainment.

[B19-jintelligence-10-00024] Harris-Watson Alexandra M., Kung Mei-Chuan, Tocci Michael C., Boyce Anthony S., Weekley Jeff A., Guenole Nigel, Carter Nathan T. (2022). The Interaction Between Conscientiousness and General Mental Ability: Support for a Compensatory Interaction in Task Performance. Journal of Business and Psychology.

[B20-jintelligence-10-00024] Hunter John E., Schmidt Frank L., Judiesch Michael K. (1990). Individual Differences in Output Variability as a Function of Job Complexity. Journal of Applied Psychology.

[B21-jintelligence-10-00024] Inácio Filomena, Faísca Luís, Forkstam Christian, Araújo Susana, Bramão Inês, Reis Alexandra, Petersson Karl Magnus (2018). Implicit sequence learning is preserved in dyslexic children. Annals of Dyslexia.

[B22-jintelligence-10-00024] Kalra Priya B., Gabrieli John D. E., Finn Amy S. (2019). Evidence of stable individual differences in implicit learning. Cognition.

[B23-jintelligence-10-00024] Kaufman Scott Barry, DeYoung Colin G., Gray Jeremy R., Jiménez Luis, Brown Jamie, Mackintosh Nicholas (2010). Implicit learning as an ability. Cognition.

[B25-jintelligence-10-00024] Kidd Evan (2012). Implicit statistical learning is directly associated with the acquisition of syntax. Developmental Psychology.

[B24-jintelligence-10-00024] Kidd Evan, Arciuli Joanne (2016). Individual differences in statistical learning predict children’s comprehension of syntax. Child Development.

[B26-jintelligence-10-00024] Knight Craig (2017). The History of Psychometrics. Psychometric Testing: Critical Perspectives.

[B27-jintelligence-10-00024] Knowlton Barbara J., Squire Larry R., Gluck Mark A. (1994). Probabilistic classification learning in amnesia. Learning & Memory.

[B28-jintelligence-10-00024] Kuhn Deanna (2016). Learning is the key twenty-first century skill. Learning: Research and Practice.

[B29-jintelligence-10-00024] Kuncel Nathan R., Hezlett Sarah A., Ones Deniz S. (2004). Academic performance, career potential, creativity, and job performance: Can one construct predict them all?. Journal of Personality and Social Psychology.

[B30-jintelligence-10-00024] Landers Richard N., Armstrong Michael B., Collmus Andrew B., Mujcic Salih, Blaik Jason (2021). Theory-driven game-based assessment of general cognitive ability: Design theory, measurement, prediction of performance, and test fairness. Journal of Applied Psychology.

[B31-jintelligence-10-00024] Mackintosh Nicholas (2004). IQ and Human Intelligence.

[B32-jintelligence-10-00024] McLeod Poppy Lauretta, Lobel Sharon Alisa, Cox Taylor H. (1996). Ethnic diversity and creativity in small groups. Small Group Research.

[B33-jintelligence-10-00024] Moses Michele S. (2010). Moral and instrumental rationales for affirmative action in five national contexts. Educational Researcher.

[B34-jintelligence-10-00024] Mount Michael K., Barrick Murray R., Stewart Greg L. (1998). Five-Factor Model of personality and Performance in Jobs Involving Interpersonal Interactions. Human Performance.

[B35-jintelligence-10-00024] Murphy Kevin R. (1989). Is the relationship between cognitive ability and job performance stable over time?. Human Performance.

[B36-jintelligence-10-00024] Neisser Ulric, Boodoo Gwyneth, Bouchard Thomas J., Boykin A. Wade, Brody Nathan, Ceci Stephen J., Halpern Diane F., Loehlin John C., Perfloff Robert, Sternberg Robert J. (1996). Intelligence: Knowns and unknowns. American Psychologist.

[B37-jintelligence-10-00024] Ng Eddy S. W., Sears Greg J. (2010). The effect of adverse impact in selection practices on organizational diversity: A field study. The International Journal of Human Resource Management.

[B38-jintelligence-10-00024] Nissen Mary Jo, Bullemer Peter (1987). Attentional requirements of learning: Evidence from performance measures. Cognitive Psychology.

[B39-jintelligence-10-00024] Noe Raymond A., Clarke Alena D. M., Klein Howard J. (2014). Learning in the twenty-first-century workplace. Annual Review of Organizational Psychology and Organizational Behavior.

[B40-jintelligence-10-00024] Nonaka Ikujiro, Toyama Ryoko (2003). The knowledge-creating theory revisited: Knowledge creation as a synthesizing process. The Essentials of Knowledge Management.

[B41-jintelligence-10-00024] Nye Christopher D., Su Rong, Rounds James, Drasgow Fritz (2012). Vocational Interests and Performance: A Quantitative Summary of Over 60 Years of Research. Perspectives on Psychological Science.

[B42-jintelligence-10-00024] Oakes David W., Ferris Gerald R., Martocchio Joseph J., Buckley M. Ronald, Broach Dana (2001). Cognitive ability and personality predictors of training program skill acquisition and job performance. Journal of Business and Psychology.

[B43-jintelligence-10-00024] Ones Deniz S., Viswesvaran Chockalingam, Schmidt Frank L. (1995). Integrity tests: Overlooked facts, resolved issues, and remaining questions. American Psychologist.

[B44-jintelligence-10-00024] Ones Deniz S., Dilchert Stephan, Viswesvaran Chockalingam, Salgado Jesús F. (2017). Cognitive ability: Measurement and validity for employee selection. Handbook of Employee Selection.

[B45-jintelligence-10-00024] Posner Michael I., Keele Steven W. (1968). On the genesis of abstract ideas. Journal of Experimental Psychology.

[B46-jintelligence-10-00024] Rathus Jill H., Reber Arthur S., Manza Louis, Kushner Michael (1994). Implicit and explicit learning: Differential effects of affective states. Perceptual and Motor Skills.

[B47-jintelligence-10-00024] Reber Arthur S. (1967). Implicit learning of artificial grammars. Journal of Verbal Learning and Verbal Behavior.

[B48-jintelligence-10-00024] Reber Arthur S. (1989). Implicit Learning and Tacit Knowledge. Journal of Experimental Psychology: General.

[B49-jintelligence-10-00024] Reber Arthur S., Walkenfeld Faye F., Hernstadt Ruth (1991). Implicit and explicit learning: Individual differences and IQ. Journal of Experimental Psychology: Learning, Memory, and Cognition.

[B50-jintelligence-10-00024] Ree Malcolm James, Earles James A. (1991). Predicting training success: Not much more than g. Personnel Psychology.

[B51-jintelligence-10-00024] Richardson Ken, Norgate Sarah H. (2015). Does IQ really predict job performance?. Applied Developmental Science.

[B52-jintelligence-10-00024] Ritchie Stuart J., Tucker-Drob Elliot M. (2018). How much does education improve intelligence? A meta-analysis. Psychological Science.

[B53-jintelligence-10-00024] Sackett Paul R., Schmitt Neal, Ellingson Jill E., Kabin Melissa B. (2001). High-stakes testing in employment, credentialing, and higher education: Prospects in a post-affirmative-action world. American Psychologist.

[B54-jintelligence-10-00024] Salgado Jesús F., Moscoso Silvia (2019). Meta-analysis of the validity of general mental ability for five performance criteria: Hunter and hunter 1984 revisited. Frontiers in Psychology.

[B55-jintelligence-10-00024] Salgado Jesús F., Anderson Neil, Moscoso Silvia, Bertua Cristina, De Fruyt Filip, Rolland Jean Pierre (2003). A meta-analytic study of general mental ability validity for different occupations in the European community. Journal of Applied Psychology.

[B56-jintelligence-10-00024] Schmidt Frank L. (2002). The role of general cognitive ability and job performance: Why there can be no debate. Human Performance.

[B59-jintelligence-10-00024] Schmidt Frank L., Hunter John (2004). General Mental Ability in the World of Work: Occupational Attainment and Job Performance. Journal of Personality and Social Psychology.

[B57-jintelligence-10-00024] Schmidt Frank L., Hunter John E. (1998). The Validity and Utility of Selection Methods in Personnel Psychology: Practical and Theoretical Implications of 85 Years of Research Findings. Psychological Bulletin.

[B58-jintelligence-10-00024] Schmidt Frank L., Hunter John E. (2003). History, Development, Evolution, and Impact of Validity Generalization and Meta-Analysis Methods, 1975–2001. Validity Generalization: A Critical Review.

[B61-jintelligence-10-00024] Schmidt Frank L., Hunter John E., Outerbridge Alice N., Goff Stephen (1988). Joint relation of experience and ability with job performance: Test of three hypotheses. Journal of Applied Psychology.

[B60-jintelligence-10-00024] Schmidt Frank L., Hunter John E., Outerbridge Alice N. (1986). Impact of job experience and ability on job knowledge, work sample performance, and supervisory ratings of job performance. Journal of Applied Psychology.

[B62-jintelligence-10-00024] Schmidt Frank L., Shaffer Jonathan A., Oh In-Sue (2008). Increased accuracy for range restriction corrections: Implications for the role of personality and general mental ability in job and training performance. Personnel Psychology.

[B63-jintelligence-10-00024] Siegelman Noam, Bogaerts Louisa, Frost Ram (2017). Measuring individual differences in statistical learning: Current pitfalls and possible solutions. Behavior Research Methods.

[B64-jintelligence-10-00024] Spearman Charles (1927). The Abilities of Man.

[B65-jintelligence-10-00024] Van Iddekinge Chad H., Aguinis Herman, Mackey Jeremy D., DeOrtentiis Philip S. (2018). A meta-analysis of the interactive, additive, and relative effects of cognitive ability and motivation on performance. Journal of Management.

[B66-jintelligence-10-00024] von Koss Torkildsen Janne, Arciuli Joanne, Wie Ona Bø (2019). Individual differences in statistical learning predict children’s reading ability in a semi-transparent orthography. Learning and Individual Differences.

